# Validation of the AUDIT-C in adults seeking help with their drinking online

**DOI:** 10.1186/s13722-016-0066-5

**Published:** 2017-01-04

**Authors:** Zarnie Khadjesari, Ian R. White, Jim McCambridge, Louise Marston, Paul Wallace, Christine Godfrey, Elizabeth Murray

**Affiliations:** 1 Department of Primary Care and Population Health, UCL Royal Free Campus, Upper Third Floor, Rowland Hill Street, London, NW3 2PF UK; 2 Health Service and Population Research Department, Centre for Implementation Science, Institute of Psychiatry, Psychology and Neuroscience, King’s College London, De Crespigny Park, London, SE5 8AF UK; 3 MRC Biostatistics Unit, Cambridge Institute of Public Health, Forvie Site, Robinson Way, Cambridge Biomedical Campus, Cambridge, CB2 0SR UK; 4 Department of Health Sciences, Seebohm Rowntree Building, University of York, Heslington, York, YO10 5DD UK

**Keywords:** Alcohol, AUDIT-C, Validation, Past week drinking, Online

## Abstract

**Background:**

The abbreviated Alcohol Use Disorder Identification Test for Consumption (AUDIT-C) is rapidly becoming the alcohol screening tool of choice for busy practitioners in clinical settings and by researchers keen to limit assessment burden and reactivity. Cut-off scores for detecting drinking above recommended limits vary by population, setting, country and potentially format. This validation study aimed to determine AUDIT-C thresholds that indicated risky drinking among a population of people seeking help over the Internet.

**Method:**

The data in this study were collected in the pilot phase of the Down Your Drink trial, which recruited people seeking help over the Internet and randomised them to a web-based intervention or an information-only website. Sensitivity, specificity, and positive and negative likelihood ratios were calculated for AUDIT-C scores, relative to weekly consumption that indicated drinking above limits and higher risk drinking. Receiver-operating characteristic (ROC) curves were created to assess the performance of different cut-off scores on the AUDIT-C for men and women. Past week alcohol consumption was used as the reference-standard and was collected via the TOT-AL, a validated online measure of past week drinking.

**Results:**

AUDIT-C scores were obtained from 3720 adults (2053 female and 1667 male) searching the internet for help with drinking, mostly from the UK. The area under the ROC curve for risky drinking was 0.84 (95% CI 0.80, 0.87) (female) and 0.80 (95% CI 0.76, 0.84) (male). AUDIT-C cut-off scores for detecting risky drinking that maximise the sum of sensitivity and specificity were ≥8 for women and ≥8 for men; whereas those identifying the highest proportion of correctly classified individuals were ≥4 for women and ≥5 for men. AUDIT-C cut-off scores for detecting higher risk drinking were also calculated.

**Conclusions:**

AUDIT-C cut-off scores for identifying alcohol consumption above weekly limits in this largely UK based study population were substantially higher than those reported in other validation studies. Researchers and practitioners should select AUDIT-C cut-off scores according to the purpose of identifying risky drinkers and hence the relative importance of sensitivity and/or specificity.

## Background

Early identification of people drinking at risky levels followed by brief intervention is the key individual-level intervention approach for reducing alcohol intake to safer levels [1, 2], with efficacy demonstrated in a range of settings including primary care, emergency departments, higher education and the workplace [3–8]. From the 1980s onwards the World Health Organisation (WHO) developed the Alcohol Use Disorders Identification Test (AUDIT), a 10-item screening questionnaire for detecting hazardous, harmful and dependent drinking in primary care [9]. There is now a substantial literature demonstrating the validity of the AUDIT in settings beyond primary care, such as inpatient hospital wards, emergency departments, universities, workplaces, outpatient settings and psychiatric services [10]. Above the basic threshold score of 8, the AUDIT guidance offers cut-off scores that indicate the severity of a person’s drinking, which in turn can be matched to the help they require, i.e. simple advice (score 8–15), simple advice plus brief counselling and continued monitoring (score 16–19), or referral to a specialist for assessment and treatment (score 20–40) [9]. These higher cut-offs are based on expert opinion rather than validation data.

Since the development of the AUDIT there have been a number of abbreviated versions that allow screening to take place in busy environments where time is limited [11]. The AUDIT-C is an abbreviated version of the AUDIT that has been advocated for use in both research and practice settings where there is insufficient time to administer the full AUDIT [11]. It consists of the first three questions of the AUDIT that relate to alcohol intake, where ‘C’ indicates ‘Consumption’ [12]. The AUDIT-C demonstrates similar accuracy to the full AUDIT [13, 14], however, the cut-off scores used to identify risky drinking, i.e. consumption above recommended limits, have varied in previous studies.

In 2007, a review of abbreviated versions of the AUDIT recommended an AUDIT-C cut-off score of ≥3 (women) and ≥4 (men) for detecting hazardous or harmful drinking [13]. This recommendation was based on a narrative review of 10 studies, of which four were in primary care patients, two in veteran populations, two in the general population [15, 16], one in hospitalised patients and one in psychiatric patients. Two studies included in this review found ‘optimal’ AUDIT-C scores (defined as those that maximise the sum of sensitivity and specificity) for detecting drinking above recommended limits in the general population of ≥5 in Germany [15] and ≥5 (men) and ≥3 (women) in the US [16]. Another review published the following year, identified four studies that tested the accuracy (i.e. the highest overall proportion of true positives and false negatives) of the AUDIT-C in detecting risky drinking in European general population samples, with cut-off scores of ≥5 and ≥6 [15, 17–19], where prevalence ranged from 5 to 37% [14]. Surprisingly few studies published since these reviews have validated the AUDIT-C in general population samples. One recently published study based in the adult general population in Sweden found the ‘optimal’ AUDIT-C cut-off score for detecting drinking above recommended limits (termed “risk drinking”) was ≥6 (men) and ≥4 (women) [20]. The AUDIT-C has not been validated for identifying risky drinking in adults from the United Kingdom.

There may be many reasons for the heterogeneity in findings in previous studies including differences in populations, settings and cultures, where both prevalence and recommended drinking limits vary. Validation studies use different reference standards and forms of measurement for determining hazardous or harmful drinking, e.g. time-line follow-back, 10-item AUDIT, International Classification of Diseases (ICD-10 criteria) and the Diagnostic and Statistical Manual of Mental Disorders (DSM-III-R, DSM-IV) [13, 14]. There are also differences in the type of cut-off scores selected, depending on the use to which the test is put.

Screening and brief intervention delivered over the Internet has grown in popularity over the past decade and is now a substantial field of research [21]. Electronic screening enables instantaneous data collection and eliminates the need for manual data entry, thereby reducing errors that this process may introduce. Alcohol screening tests, which are conventionally delivered in-person or in paper-based format, appear to retain their psychometric properties when delivered online [22–26]. There is also some evidence that being self-administered, online screening is likely to generate more honest reporting of risky alcohol use, in comparison with a face-to-face interview [27, 28]. The AUDIT-C has been used to screen for eligibility in two trials of web-based alcohol screening and brief intervention delivered to students in New Zealand (≥4 for men and women) [29, 30] and two trials of facilitated access to an online intervention delivered in primary care in Italy and Spain (≥5 for men and ≥4 women) [31, 32]. These trials did not validate the AUDIT-C for use online, and were not conducted in general population samples.

The purpose of this study was to determine a suitable cut-off score for the AUDIT-C for identifying risky drinkers in a general population sample of people seeking online help with their drinking. Objectives were to determine the sensitivity, specificity, likelihood ratios and area under the Receiver-operating characteristic (ROC) curves of different cut-off scores for the AUDIT-C, with a goal of identifying people drinking above the recommended UK weekly consumption limits. To the best of our knowledge, this is the first study that seeks to validate the AUDIT-C in a population of people seeking help with their drinking over the Internet.

## Methods

The data for this study were collected during the eight month pilot phase (February to October 2007) of an online randomised controlled trial investigating the effectiveness of an internet-based intervention (called Down Your Drink—DYD [33] for people looking for help or information on their drinking [34, 35]. One of the objectives of this pilot trial was to determine a suitable AUDIT-C cut-off score for identifying people drinking above UK weekly limits advocated by the Royal Colleges of General Practitioners and Psychiatrists and Department of Health [36, 37] for use in recruiting to the main trial phase of Down Your Drink [34]. Ethical approval for the DYD pilot trial was obtained from UCL Research Ethics Committee. The Down Your Drink website was identified via Internet searches for help or information on drinking, or from the home page of Alcohol Concern, the UK’s largest alcohol charity; no further advertising was needed to meet the sample size for the pilot trial. The DYD homepage asked visitors to “find out if you are drinking too much” by directing them to the AUDIT-C questionnaire. In order to gain access to the Down Your Drink website, people were required to enter an online trial and provide informed consent, if aged 18 years or above. Visitors subsequently registered with the website and completed baseline data before being randomised. The first baseline questionnaire, following the initial screen with the AUDIT-C, was an online measure of past week drinking (the TOT-AL, detailed below), which was followed by other validated measures of alcohol problems and dependence [34].

### AUDIT-C

The AUDIT-C constitutes the following three questions:

1. How often do you have a drink containing alcohol? Answer: Never (score 0), Monthly or less (score 1), 2–4 times per month (score 2), 2–3 times per week (score 3), 4+ times per week (score 4);
2. How many units of alcohol do you drink on a typical day when you are drinking? Answer: 1–2 (score 0), 3–4 (score 1), 5–6 (score 2), 7–9 (score 3), 10+ (score 4);
3. How often have you had 6 or more units if female, or 8 or more if male, on a single occasion in the last year? Answer: Never (score 0), Less than monthly (score 1), Monthly (score 2), Weekly (score 3), Daily or almost daily (score 4).

### Reference standard

Our reference standard is the TOT-AL measure of past week drinking, which is used here to identify two conditions: (1) risky drinking, and (2) higher risk drinking.

1. Weekly drinking limits recommended by the Royal Colleges of General Practitioners and Psychiatrists [36] and previously by the Department of Health [37] were used as the reference standards to evaluate the performance of the AUDIT-C:
  14 units of alcohol per week for women;
  21 units of alcohol per week for men, where (1 UK unit is 8 grams of ethanol). At the time of writing there was a consultation on reducing this to 14 units [38].
2. We were also interested in evaluating performance against the accepted UK threshold for a level of heavy drinking at which problems are likely to be occurring. These “higher risk” thresholds were:
  35 units of alcohol per week for women;
  50 units of alcohol per week for men.

The TOT-AL is a reliable and valid online measure that presents drop-down menus on the type, brand, size and quantity of alcohol consumed on each of the past seven days and calculates total units of alcohol consumed (measured in UK units) [39]. There is a strong correlation between repeated measurements of the TOT-AL (r = 0.99; 95% CI 0.98, 0.99) and between the units calculated by the TOT-AL and a face-to face interview (r = 0.97; 95% CI 0.95, 0.99). A high level of agreement between measurements was also observed in a Bland–Altman analysis [39]. The TOT-AL was completed by all participants. Data were entered anonymously by participants from a computer with Internet access from any location.

### Analyses

The sensitivity and specificity of cut-off scores between two and ten on the AUDIT-C were examined separately for males and females using the recommended weekly drinking limits (measured by the TOT-AL) as the reference-standard. Positive and negative likelihood ratios were calculated to estimate how different cut-off scores change the odds of being a risky drinker and a higher risk drinker, where positive likelihood ratios = sensitivity/(1 − specificity), and negative likelihood ratios = (1 − sensitivity)/specificity. Receiver-operating characteristic (ROC) curves were created to assess the performance of different cut-off scores on the AUDIT-C for men and women. ROC curves plot the sensitivities of different cut-off scores against 1-specificities (known as the false positive rate). The area under the ROC curve quantifies the ability of the AUDIT-C to discriminate between those people drinking above and within weekly drinking limits. A perfect test is indicated by an area under the ROC curve of 1.0, whereas a worthless test is indicated by 0.5. Analyses were conducted in Stata V13 [40].

Validation studies of the AUDIT-C in general population settings tend to report a cut-off score that maximises the sum of sensitivity and specificity. We refer to this as an ‘optimal’ cut-off score [41]. This cut-off score is used when the sensitivity and specificity of a test are of equal importance. In addition to these ‘optimal’ cut-off scores, our study also presents ‘accuracy’ cut-off scores which identify the highest overall proportion of correctly classified risky and lower-risk drinkers.

When the same data are used both to select a cut-off score and to evaluate performance (sensitivity, specificity, likelihood ratios or accuracy) at the cut-off, performance tends to be over-estimated—a phenomenon known as “optimism” or “overfitting” [42]. To avoid this, the data were randomly split into two subsets. The cut-offs were re-estimated in one subset and their performance was evaluated in the other subset, and vice versa, and the estimated performances were averaged to give an “optimism-adjusted” performance. This procedure was not needed in evaluating performance at fixed cut-offs.

## Results

### Baseline characteristics

**Fig. 1:**
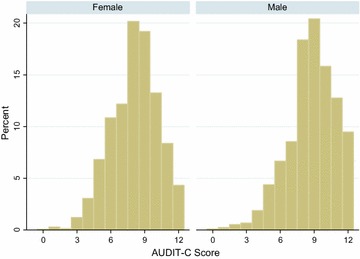
Distribution of AUDIT-C scores in female and male participants

A total of 3720 participants completed baseline measures in the pilot trial. All participants included in this study had data for both TOT-AL and AUDIT-C measures, there was no drop out or withdrawals. Participants were mostly female (55%), with an average age of 37 years (SD 11), mostly ‘White British’ (84%) and living in the UK (89%). Participants living outside the UK were most commonly from other Anglophone countries (highest U.S. n = 108, Canada n = 40) with small numbers from other countries (highest France n = 28). Half of all participants were educated to university degree level or above (51%). Average (geometric mean − given the skewed distribution of the data) alcohol intake at baseline was 38 UK units in the past week (SD 4) and the mean AUDIT-C score was 8 (SD 2), for distribution of AUDIT-C scores in men and women see Fig. 1. The mean number of drinking days in past week was 5 (SD 2) and mean number of days drinking >6 ♀/>8 ♂ units of alcohol in past week, was 3 (SD 2). Baseline characteristics are reported separately by gender in Table 1.

**Table 1:** Demographics

Demographic variables	Female N = 2053	Male N = 1667
Age (years): mean (SD)	37 (11)	38 (11)
Educated at least to degree level: n (%)	1039 (50)	853 (51)
White British: n (%)	1785 (86)	1363 (82)
Living in UK: n (%)	1883 (91)	1439 (86)
AUDIT-C: mean (SD)	8 (2)	9 (2)
Past week’s alcohol consumption in units: arithmetic mean (SD)^a^	48 (30)	64 (42)
Past week’s alcohol consumption in units: geometric mean (approx. SD^b^)^a^	35 (4)	43 (5)
Number of drinking days in past week: mean (SD)^a^	5 (2)	5 (2)
Number of days drinking >6 ♀/>8 ♂ units of alcohol in past week: mean (SD)^a^	3 (2)	3 (2)
Drinking >14 ♀/>21 ♂ units of alcohol in past week: n (%)^a,c^	1901 (91)	1486 (89)
Drinking >35 ♀/>50 ♂ units of alcohol in past week: n (%)^a,d^	1298 (62)	937 (56)

^a^Drinking measures using data collected by the TOT-AL

^b^Approximate SD back-calculated from the log-scale

^c^Above weekly limits

^d^Higher risk drinking

### Drinking above recommended weekly limits

**Fig. 2:**
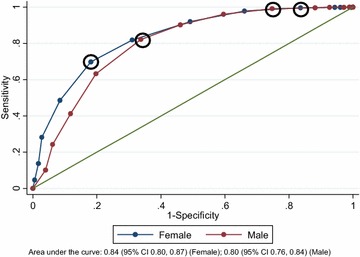
Receiver-operating characteristic curve for drinking above weekly limits

The area under the ROC curve was 0.84 (95% CI 0.80, 0.87) for females and 0.80 (95% CI 0.76, 0.84) for males (Fig. 2).

The ‘optimal’ AUDIT-C cut-off scores for identifying people drinking above weekly limits were found to be ≥8 (female) and ≥8 (male). Performance at optimal cut-offs are reported in Table 6 without and with adjustment for optimism, but the optimism-adjusted values are described here. Estimated sensitivity was 76% (95% CI 74, 78) and specificity 73% (95% CI 66, 79) for women, and sensitivity was 85% (95% CI 83, 87) and specificity of 58% (95% CI 50, 65) for men. The positive likelihood ratios corresponding to a cut-off score of ≥8 were 2.81 (95% CI 2.56, 3.08) for women and 2.02 (95% CI 1.78, 2.29) for men; the negative likelihood ratios were 0.32 (95% CI 0.25, 0.41) for women and 0.26 (95% CI 0.21, 0.32) for men (Tables 2, 3, 6).

**Table 2:** AUDIT-C threshold for drinking above weekly limits (female)

AUDIT-C score	Sensitivity % (95% CI)	Specificity % (95% CI)	LR+^a^ (95% CI)	LR−^b^ (95% CI)	Sensitivity + specificity	% Correctly classified (95% CI)
≥2	99.95 (99.70, 100.0)	3.95 (1.60, 7.98)	1.04 (0.50, 2.15)	0.01 (0.00, 0.07)	103.90	91.67 (90.39, 92.83)
≥3	99.95 (99.70, 100.0)	5.65 (2.74, 10.14)	1.06 (0.58, 1.94)	0.01 (0.00, 0.07)	105.60	91.82 (90.55, 92.97)
≥4*	99.63 (99.23, 99.85)	16.38 (11.26, 22.68)	1.19 (0.85, 1.66)	0.02 (0.01, 0.04)	116.01	92.45 (91.22, 93.56)
≥5	97.92 (97.17, 98.52)	33.90 (26.97, 41.38)	1.48 (1.20, 1.82)	0.06 (0.04, 0.08)	131.82	92.40 (91.17, 93.51)
≥6	92.06 (90.74, 93.24)	50.85 (43.24, 58.43)	1.87 (1.62, 2.16)	0.16 (0.13, 0.20)	142.91	88.50 (87.05, 89.85)
≥7	81.88 (80.06, 83.60)	68.93 (61.55, 75.66)	2.63 (2.38, 2.91)	0.26 (0.20, 0.33)	150.81	80.76 (78.99, 82.44)
≥8**	69.78 (67.64, 71.85)	81.92 (75.45, 87.29)	3.86 (3.58, 4.16)	0.37 (0.27, 0.51)	151.70	70.82 (68.80, 72.78)
≥9	48.61 (46.33, 50.90)	91.53 (86.41, 95.18)	5.74 (5.38, 6.12)	0.56 (0.34, 0.91)	140.14	52.31 (50.13, 54.49)
≥10	28.14 (26.12, 30.24)	97.18 (93.53, 99.08)	9.96 (9.23, 10.75)	0.74 (0.31, 1.76)	125.32	34.10 (32.05, 36.19)

Drinking >14 units of alcohol in past week

*The most accurate AUDIT-C cut-off score for identifying risky drinkers

**The ‘optimal’ AUDIT-C cut-off score for identifying risky drinkers

^a^LR+ positive likelihood ratio

^b^LR− negative likelihood ratio

**Table 3:** AUDIT-C threshold for drinking above weekly limits (male)

AUDIT-C score	Sensitivity % (95% CI)	Specificity % (95% CI)	LR+^a^ (95% CI)	LR−^b^ (95% CI)	Sensitivity + specificity	% Correctly classified (95% CI)
≥2	99.93 (99.62, 100.0)	2.81 (0.92, 6.43)	1.03 (0.43, 2.44)	0.02 (0.00, 0.14)	102.74	89.35 (87.75, 90.81)
≥3	99.86 (99.50, 99.98)	7.30 (3.95, 12.17)	1.08 (0.64, 1.82)	0.02 (0.01, 0.08)	107.16	89.78 (88.21, 91.21)
≥4	99.66 (99.20, 99.89)	11.80 (7.45, 17.47)	1.13 (0.76, 1.69)	0.03 (0.01, 0.07)	111.46	90.09 (88.53, 91.49)
≥5*	99.18 (98.56, 99.57)	25.28 (19.08, 32.33)	1.33 (1.03, 1.71)	0.03 (0.02, 0.05)	124.46	91.13 (89.64, 92.46)
≥6	96.09 (94.96, 97.02)	40.45 (33.17, 48.05)	1.61 (1.35, 1.92)	0.10 (0.08, 0.13)	136.54	90.02 (88.47, 91.44)
≥7	90.25 (88.61, 91.72)	53.93 (46.32, 61.42)	1.96 (1.71, 2.25)	0.18 (0.14, 0.22)	144.18	86.29 (84.53, 87.92)
≥8**	82.14 (80.08, 84.08)	66.29 (58.84, 73.19)	2.44 (2.19, 2.72)	0.27 (0.21, 0.34)	148.43	80.42 (78.41, 82.31)
≥9	63.19 (60.65, 65.67)	80.34 (73.73, 85.91)	3.21 (2.96, 3.49)	0.46 (0.34, 0.62)	143.53	65.06 (62.69, 67.37)
≥10	41.21 (38.67, 43.79)	88.20 (82.53, 92.55)	3.49 (3.22, 3.79)	0.67 (0.45, 1.00)	129.41	46.33 (43.89, 48.78)

Drinking >21 units of alcohol in past week

*The most accurate AUDIT-C cut-off score for identifying risky drinkers

**The ‘optimal’ AUDIT-C cut-off score for identifying risky drinkers

^a^LR+ positive likelihood ratio

^b^LR− negative likelihood ratio

The most accurate AUDIT-C cut-off scores for identifying people drinking above weekly limits were ≥4 (female) and ≥5 (male), with corresponding sensitivity of 99% (95% CI 98, 99) and specificity of 25% (95% CI 19, 33) for females, and sensitivity of 99% (95% CI 99, 100) and specificity of 25% (95% CI 19, 32) for males. These cut-off scores led to a high proportion of participants correctly identified as drinking above recommended limits for both females (92%) and males (91%). The positive likelihood ratios corresponding to a cut-off score of ≥4 was 1.32 (95% CI 1.03, 1.70) for women and ≥5 was 1.33 (95% CI 1.03, 1.71) for men; the negative likelihood ratios were 0.05 (95% CI 0.03, 0.07) for women and 0.03 (95% CI 0.02, 0.05) for men (Tables 2, 3, 6).

### Higher risk drinking

**Fig. 3:**
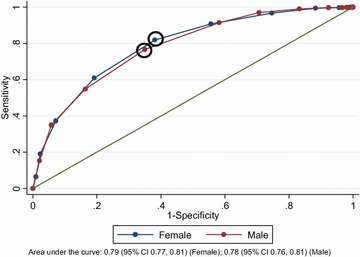
Receiver-operating characteristic curve for higher risk drinking

The area under the ROC curve was 0.79 (95% CI 0.77, 0.81) for females and 0.78 (95% CI 0.76, 0.81) for males (Fig. 3).

The ‘optimal’ AUDIT-C cut-off scores for identifying higher risk drinkers, i.e. more than 35 units/week for women and more than 50 units/week for men) was found to be ≥8 for women and ≥9 for men, with corresponding sensitivity of 70% (95% CI 67, 73) and specificity of 71% (95% CI 67, 74) for women, and sensitivity of 77% (95% CI 74, 79) and specificity of 65% (95% CI 61, 69) for men. The positive likelihood ratios corresponding to a cut-off score of ≥8 was 2.39 (95% CI 2.26, 2.53) for women and ≥9 was 2.19 (95% CI 2.05, 2.34) for men; the negative likelihood ratios were 0.42 (95% CI 0.37, 0.48) for women and 0.36 (95% CI 0.31, 0.42) for men (Tables 4, 5, 6).

**Table 4:** AUDIT-C thresholds for higher risk drinking (female)

AUDIT-C score	Sensitivity % (95% CI)	Specificity % (95% CI)	LR+^a^ (95% CI)	LR−^b^ (95% CI)	Sensitivity + specificity	% Correctly classified (95% CI)
≥2	99.92 (99.56, 100.0)	0.90 (0.36, 1.85)	1.01 (0.48, 2.11)	0.09 (0.01, 0.64)	100.82	62.45 (60.31, 64.55)
≥3	99.92 (99.56, 100.0)	1.29 (0.62, 2.35)	1.01 (0.55, 1.87)	0.06 (0.02, 0.24)	101.21	62.59 (60.46, 64.69)
≥4	99.76 (99.31, 99.95)	4.25 (2.94, 5.91)	1.04 (0.74, 1.45)	0.06 (0.02, 0.19)	104.01	63.61 (61.49, 65.70)
≥5	99.37 (98.77, 99.73)	11.71 (9.54, 14.18)	1.13 (0.93, 1.37)	0.05 (0.03, 0.10)	111.08	66.20 (64.10, 68.24)
≥6	96.71 (95.58, 97.62)	25.35 (22.33, 28.57)	1.30 (1.15, 1.47)	0.13 (0.10, 0.18)	122.06	69.70 (67.66, 71.69)
≥7	90.83 (89.11, 92.36)	44.40 (40.87, 47.98)	1.63 (1.50, 1.77)	0.21 (0.17, 0.25)	135.23	73.26 (71.29, 75.16)
≥8*	81.90 (79.67, 83.97)	61.90 (58.39, 65.33)	2.15 (2.02, 2.28)	0.29 (0.25, 0.34)	143.80	74.33 (72.38, 76.21)
≥9	60.97 (58.23, 63.66)	80.82 (77.88, 83.53)	3.18 (3.01, 3.36)	0.48 (0.41, 0.56)	141.79	68.49 (66.43, 70.49)
≥10	37.38 (34.72, 40.10)	92.79 (90.74, 94.51)	5.19 (4.82, 5.59)	0.67 (0.52, 0.87)	130.17	58.35 (56.19, 60.50)

Drinking >35 units of alcohol in past week

*The ‘optimal’ and most accurate AUDIT-C cut-off score for identifying higher risk drinkers

^a^LR+ positive likelihood ratio

^b^LR− negative likelihood ratio

**Table 5:** AUDIT-C thresholds for higher risk drinking (male)

AUDIT-C score	Sensitivity % (95% CI)	Specificity % (95% CI)	LR+^a^ (95% CI)	LR−^b^ (95% CI)	Sensitivity + specificity	% Correctly classified (95% CI)
≥2	100.00 (99.60, 100.0)	0.84 (0.31, 1.82)	1.01 (0.46, 2.24)	0.00	100.84	56.61 (54.17, 59.03)
≥3	99.89 (99.40, 100.0)	1.96 (1.07, 3.26)	1.02 (0.61, 1.71)	0.06 (0.01, 0.43)	101.85	57.04 (54.60, 59.45)
≥4	99.78 (99.22, 99.97)	3.36 (2.16, 4.95)	1.03 (0.70, 1.53)	0.06 (0.02, 0.24)	103.14	57.59 (55.15, 60.00)
≥5	99.78 (99.22, 99.97)	7.69 (5.85, 9.90)	1.08 (0.84, 1.39)	0.03 (0.01, 0.12)	107.47	59.49 (57.06, 61.88)
≥6	99.02 (98.15, 99.55)	16.78 (14.12, 19.73)	1.19 (1.01, 1.40)	0.06 (0.03, 0.12)	115.8	63.04 (60.64, 65.38)
≥7	96.95 (95.63, 97.97)	29.37 (26.05, 32.86)	1.37 (1.22, 1.54)	0.10 (0.07, 0.14)	126.32	67.38 (65.05, 69.65)
≥8	91.40 (89.40, 93.14)	41.82 (38.17, 45.53)	1.57 (1.44, 1.72)	0.21 (0.17, 0.26)	133.22	69.71 (67.41, 71.93)
≥9*	76.71 (73.84, 79.41)	65.03 (61.41, 68.53)	2.19 (2.05, 2.34)	0.36 (0.31, 0.42)	141.74	71.60 (69.35, 73.78)
≥10	54.84 (51.56, 58.09)	83.64 (80.72, 86.27)	3.35 (3.13, 3.58)	0.54 (0.45, 0.65)	138.48	67.44 (65.11, 69.71)

Drinking >50 units of alcohol in past week

*The ‘optimal’ and most accurate AUDIT-C cut-off score for identifying higher risk drinkers

^a^LR+ positive likelihood ratio

^b^LR− negative likelihood ratio

**Table 6:** Optimal and most accurate AUDIT-C thresholds

Drinking category	Selected thresholds by sex, evaluations with and without optimism			Sensitivity (%) (95% CI)	Specificity (%) (95% CI)	LR+^c^ (95% CI)	LR−^d^ (95% CI)	Sensitivity + specificity	% Correctly classified (95% CI)
Above weekly limits^a^	Optimal	Female ≥8	With optimism	69.78 (67.64, 71.85)	81.92 (75.45, 87.29)	3.86 (3.58, 4.16)	0.37 (0.27, 0.51)	151.7	70.82 (68.80, 72.78)
			Optimism-adjusted	76.33 (74.34, 78.24)	72.88 (65.70, 79.28)	2.81 (2.56, 3.08)	0.32 (0.25, 0.41)	149.21	76.04 (74.13, 77.87)
		Male ≥8	With optimism	82.14 (80.08, 84.08)	66.29 (58.84, 73.19)	2.44 (2.19, 2.72)	0.27 (0.21, 0.34)	148.43	80.42 (78.41, 82.31)
			Optimism-adjusted	85.10 (83.16, 86.89)	57.87 (50.25, 65.21)	2.02 (1.78, 2.29)	0.26 (0.21, 0.32)	142.97	82.13 (80.18, 83.96)
	Most accurate	Female ≥4	With optimism	99.63 (99.23, 99.85)	16.38 (11.26, 22.68)	1.19 (0.85, 1.66)	0.02 (0.01, 0.04)	116.01	92.45 (91.22, 93.56)
			Optimism-adjusted	98.61 (97.98, 99.09)	25.42 (19.19, 32.50)	1.32 (1.03, 1.70)	0.05 (0.03, 0.07)	124.03	92.30 (91.07, 93.42)
		Male ≥5	With optimism	99.18 (98.56, 99.57)	25.28 (19.08, 32.33)	1.33 (1.03, 1.71)	0.03 (0.02, 0.05)	124.46	91.13 (89.64, 92.46)
			Optimism-adjusted	99.18 (98.56, 99.57)	25.28 (19.08, 32.33)	1.33 (1.03, 1.71)	0.03 (0.02, 0.05)	124.46	91.13 (89.64, 92.46)
Higher risk drinking^b^	Optimal	Female ≥8	With optimism	81.90 (79.67, 83.97)	61.90 (58.39, 65.33)	2.15 (2.02, 2.28)	0.29 (0.25, 0.34)	143.80	74.33 (72.38, 76.21)
			Optimism-adjusted	81.90 (79.67, 83.97)	61.90 (58.39, 65.33)	2.15 (2.02, 2.28)	0.29 (0.25, 0.34)	143.80	74.33 (72.38, 76.21)
		Male ≥9	With optimism	76.71 (73.84, 79.41)	65.03 (61.41, 68.53)	2.19 (2.05, 2.34)	0.36 (0.31, 0.42)	141.74	71.60 (69.35, 73.78)
			Optimism-adjusted	76.71 (73.84, 79.41)	65.03 (61.41, 68.53)	2.19 (2.05, 2.34)	0.36 (0.31, 0.42)	141.74	71.60 (69.35, 73.78)
	Most accurate	Female ≥8	With optimism	81.90 (79.67, 83.97)	61.90 (58.39, 65.33)	2.15 (2.02, 2.28)	0.29 (0.25, 0.34)	143.80	74.33 (72.38, 76.21)
			Optimism-adjusted	81.90 (79.67, 83.97)	61.90 (58.39, 65.33)	2.15 (2.02, 2.28)	0.29 (0.25, 0.34)	143.80	74.33 (72.38, 76.21)
		Male ≥9	With optimism	76.71 (73.84, 79.41)	65.03 (61.41, 68.53)	2.19 (2.05, 2.34)	0.36 (0.31, 0.42)	141.74	71.60 (69.35, 73.78)
			Optimism-adjusted	76.71 (73.84, 79.41)	65.03 (61.41, 68.53)	2.19 (2.05, 2.34)	0.36 (0.31, 0.42)	141.74	71.60 (69.35, 73.78)

^a^Above weekly limits >14 units of alcohol in past week (women); >21 units of alcohol in past week (men)

^b^Higher risk drinking >35 units of alcohol in past week (women); >50 units of alcohol in past week (men)

^c^LR+ positive likelihood ratio

^d^LR− negative likelihood ratio

The most accurate AUDIT-C cut-off scores for identifying higher risk drinkers were also found to be ≥8 for women and ≥9 for men (Tables 4, 5, 6). These cut-off scores identified the highest proportion of participants correctly identified as drinking at higher risk levels for both women (70%) and men (72%).

## Discussion

This study identified AUDIT-C thresholds that indicated risky and higher risk drinking among adults seeking online help with their drinking. This study found that ‘optimal’ AUDIT-C cut-off scores, defined as those that maximise the sum of sensitivity and specificity, for identifying drinking above recommended weekly limits were ≥8 for women and ≥8 for men; whereas the most accurate AUDIT-C cut-off scores, i.e. those with the highest proportion of individuals correctly classified as risky, were ≥4 for women and ≥5 for men. Optimal and accurate AUDIT-C cut-off scores for identifying higher risk drinkers were equal at ≥8 for women and ≥9 for men. These findings relate to a largely UK based population of adults seeking online help with their drinking.

The optimal cut-off scores for identifying people drinking above advocated weekly limits were substantially higher in this study of online help seekers predominantly from the UK than in other validation studies in general population samples. Online help seekers are a novel population in this field of study. Studies in the US, Germany and Scandinavia identified in the small number of reviews in this field [13, 14] have validated the AUDIT-C for detecting risky drinking in the general population, all of which administered measures in-person. The optimal AUDIT-C scores found in the present study are higher than those previously identified which is a potentially important finding, particularly for researchers evaluating the effectiveness of brief alcohol interventions accessible over the Internet, as thresholds that are set too low may underestimate intervention impact if they are not appropriately targeted.

This study included a sample of people who were web-browsing and visited the Down Your Drink site. Some, at least, will have been actively seeking help, and they may therefore display different characteristics to opportunistically-recruited non-help seeking populations in primary care and other settings in which brief intervention studies usually take place. Participants were concerned enough to think about or change their drinking. These ‘e-help’ seekers consumed higher levels of alcohol than the general (non-help seeking) population, with almost the entire sample drinking above recommended limits (91% female, 89% male). DYD participants also differ from the general population as a whole by reporting fewer problems with mobility, self-care, usual activities and pain because they were younger. However, they were more likely to report experiencing anxiety and/or depression (57% DYD vs. 21% general population) [43]. The nature of this study population thus warrants careful consideration in relation to study findings and the generalisability of these data.

When selecting a suitable threshold for the identification of risky drinkers there are various factors that need to be considered, such as prevalence, the regularity of screening, and any physical, psychological and economic costs related to the identification of false positives or false negatives [44]. For example, when screening for risky drinking in primary care settings, it has been suggested that sensitivity may be more important than specificity due to the relative ease and low cost of further assessment [12]. Contrary to this, the US Department of Veteran Affairs medical centres use an AUDIT-C threshold of ≥5 for men and women as a means of minimising burden of false positives on primary care providers, where recommended thresholds in this setting are typically lower [45]. It is important to note that the present study was conducted with an online help-seeking population and should be used to inform screening of populations identified in a similar manner. In the DYD trial, we used the most accurate cut-off scores to screen adults for risky and higher risk drinking. In this context we wanted to maximise the number of correctly screened individuals, with no particular emphasis on sensitivity—no harm was associated with false positives, and no particular emphasis on specificity—the intervention was delivered online with no time or financial restraints on its delivery. Rather, it was important that the test was credible in detecting whether people were at risk from their drinking or not. It has been suggested that ‘optimal’ cut-off scores, which maximise sensitivity and specificity, are nonsensical as they often combine accuracy data from thresholds that are not clinically relevant [46]. We suggest that researchers, practitioners and policy makers think carefully about the context and implications of alcohol screening before selecting or advocating an AUDIT-C cut-off score.

### Strengths and limitations

One of the key strengths of this study is the use of past week drinking data as the reference-standard with which the AUDIT-C scores were compared. In using a very detailed online measure of past week drinking (the TOT-AL), we were able to determine the AUDIT-C cut-off score at which participants were drinking above the recommended UK weekly limits of 14 units per week for women, and 21 units per week for men. Many of the studies investigating different cut-off scores for abbreviated versions of the AUDIT have used the full AUDIT as their reference-standard which “violates the independence of data assumption underlying the use of these statistical tests” p. 22 [26]. Furthermore, the AUDIT-C is a measure of alcohol consumption, not harm, therefore, we deemed a measure of consumption as a more suitable reference standard than a combined measure of consumption and harm.

The TOT-AL measures past week consumption, known as actual or exact recall, which leads to easier and more accurate recall due to the recency of consumption, and avoids difficulties in attempts to estimate average consumption [41, 47]. This helps minimise task-related errors, though the nature of the behaviour being reported upon is intrinsically difficult to measure retrospectively, as recall must contend with variations in drinking patterns over time [41, 47]. In addition, short-term measurement does not accurately reflect alcohol consumption among infrequent drinkers due to inadequate time sampling [41, 47].

The use of weekly limits as a reference-standard is also arguably a weakness. Despite the focus of the AUDIT-C on consumption, it was principally developed as a screening tool for problematic drinking [12]. As such, one limitation of this study, notwithstanding the strong population-level correlation between consumption and problems, is that we are unable to classify participants as problematic drinkers, in the absence of any individual-level information on alcohol-related harm or problems. This also limits the generalisability of this study, if one is interested in identifying people who are experiencing current problems and may be more receptive to interventions than those who are not [48]. Note also that levels of consumption or compliance with weekly drinking limits per se provide information on risk (i.e. possible future problems), whereas the AUDIT was originally designed as a clinical instrument concerned with need for brief intervention [9]. Further limitations include the small student sample in which the TOT-AL was validated, and that past-week consumption may not reflect average consumption, and therefore differs from the AUDIT-C in that respect.

This study did not investigate whether different AUDIT-C scores were necessary for identifying risky drinking in different age groups as participants were aged 18 years or above, and due to the online nature of the DYD trial, older people may have been under-represented. Previous research has found that lower cut-off scores may be necessary in younger age groups [49], and the Royal College of Psychiatrists have advocated that lower recommended limits are introduced for people over the age of 65 [50]. Previous research findings are mixed as to the need for different cut-off scores for different ethnicities [51, 52]. This study constituted a largely ‘White British’ population (84%), therefore exploration of suitability for different ethnic groups was not possible.

## Conclusion

The ‘optimal’ AUDIT-C scores for identifying people drinking above recommended weekly drinking limits were substantially higher in this study than in any previous study undertaken in any form of general population sample. This is one of the few studies that has validated the AUDIT-C in an adult UK population, and the web browsing nature of this sample is emphasised in interpreting these data. Researchers should consider carefully the basis for selecting AUDIT-C cut-off scores according to the purposes of identifying risky drinkers, the relative importance of sensitivity and/or specificity, and the setting in which screening is undertaken.
